# A Sephin1-insensitive tripartite holophosphatase dephosphorylates translation initiation factor 2α

**DOI:** 10.1074/jbc.RA118.002325

**Published:** 2018-04-04

**Authors:** Ana Crespillo-Casado, Zander Claes, Meng S. Choy, Wolfgang Peti, Mathieu Bollen, David Ron

**Affiliations:** From the ‡ Cambridge Institute for Medical Research, University of Cambridge, Cambridge CB2 0XY, United Kingdom,; the § Department of Cellular and Molecular Medicine, KU Leuven, 3000 Leuven, Belgium, and; the ¶ Department of Chemistry and Biochemistry, University of Arizona, Tucson, Arizona 85721-0041

**Keywords:** phosphoprotein phosphatase 1 (PP1), eukaryotic initiation factor 2 (eIF2), enzyme inhibitor, G-actin, proteostasis, guanabenz, integrated stress response, protein synthesis, Sephin1

## Abstract

The integrated stress response (ISR) is regulated by kinases that phosphorylate the α subunit of translation initiation factor 2 and phosphatases that dephosphorylate it. Genetic and biochemical observations indicate that the eIF2α^P^-directed holophosphatase, a therapeutic target in diseases of protein misfolding, is comprised of a regulatory subunit, PPP1R15, and a catalytic subunit, protein phosphatase 1 (PP1). In mammals, there are two isoforms of the regulatory subunit, PPP1R15A and PPP1R15B, with overlapping roles in the essential function of eIF2α^P^ dephosphorylation. However, conflicting reports have appeared regarding the requirement for an additional co-factor, G-actin, in enabling substrate-specific dephosphorylation by PPP1R15-containing PP1 holoenzymes. An additional concern relates to the sensitivity of the holoenzyme to the [(o-chlorobenzylidene)amino]guanidines Sephin1 or guanabenz, putative small-molecule proteostasis modulators. It has been suggested that the source and method of purification of the PP1 catalytic subunit and the presence or absence of an N-terminal repeat–containing region in the PPP1R15A regulatory subunit might influence the requirement for G-actin and sensitivity of the holoenzyme to inhibitors. We found that eIF2α^P^ dephosphorylation by PP1 was moderately stimulated by repeat-containing PPP1R15A in an unphysiological low ionic strength buffer, whereas stimulation imparted by the co-presence of PPP1R15A and G-actin was observed under a broad range of conditions, low and physiological ionic strength, regardless of whether the PPP1R15A regulatory subunit had or lacked the N-terminal repeat–containing region and whether it was paired with native PP1 purified from rabbit muscle or recombinant PP1 purified from bacteria. Furthermore, none of the PPP1R15A-containing holophosphatases tested were inhibited by Sephin1 or guanabenz.

## Introduction

The integrated stress response (ISR)^6^ is a signal transduction pathway that couples diverse stressful conditions to the activation of a rectifying translational and transcriptional program that is implicated in biological processes ranging from memory formation to immunity and metabolism (reviewed in Ref. 1). The mammalian ISR and its yeast counterpart (the general control response) are initiated by phosphorylation of the α subunit of translation initiation factor 2 (eIF2α) on serine 51 (2, 3), and its activity is terminated by eIF2α^P^ dephosphorylation.

**Figure 1.**
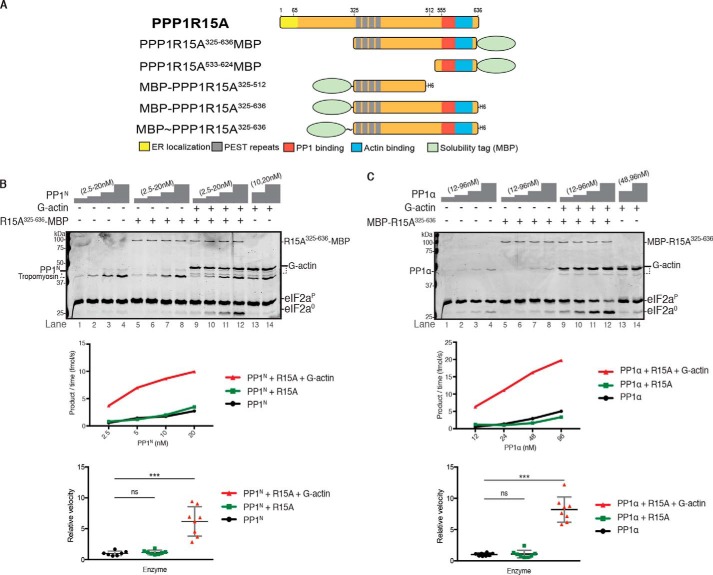
**G-actin stimulates PPP1R15A-dependent eIF2αP dephosphorylation by either PP1N or PP1α.** *A*, cartoon representation of human PPP1R15A protein (1–674) and the different constructs used in this study (sequence provided in Table S1). Key residues used for truncated versions of the proteins in this study are annotated. The ER localization domain and the proline, glutamate, serine, and threonine-rich (*PEST*) repeats are highlighted, as are the PP1 and G-actin binding sites in the conserved C-terminal region. The MBP solubility tag is also represented in the cartoons of the constructs. *B*, *top panel*, Coomassie-stained PhosTag SDS-PAGE containing resolved samples of dephosphorylation reactions (30 min at 30 °C) in which 2 μm eIF2α^P^ was dephosphorylated by PP1^N^ purified from rabbit skeletal muscle in the presence or absence of PPP1R15A^325–636^-MBP (50 nm) and/or G-actin (400 nm).The position of the various protein species is indicated. eIF2α^P^ and eIF2α^0^ refer to the phosphorylated and nonphosphorylated form of the bacterially expressed N-terminal domain (residues 1–185) of eIF2α, respectively. Note that both G-actin and PP1^N^ preparation gave rise to two bands: a major full-length species and minor degradation product in the case of G-actin and a PP1and tropomyosin band in the case of PP1^N^ (see also Fig. S1). Shown is a representative experiment of two independent repetitions performed. *Center panel*, plot of the rate of eIF2α^P^ dephosphorylation as a function of the concentration of PP1^N^ from *lanes 1–12* of the experiment above. *Bottom panel*, plot of the velocity of each enzyme relative to the mean of velocity of PP1 alone calculated from all the informative reactions in the two repeats of this experiment. Statistical significance was derived from Mann-Whitney test (*ns*, nonsignificant, *p* > 0.05; ***, *p* ≤ 0.001). *C*, as in *B* but using bacterially expressed PP1α as the catalytic subunit (96, 48, 24, or 12 nm), MBP-PPP1R15A^325–636^ (50 nm), and G-actin (400 nm). The assays were performed during 20 min at 30 °C. Shown is a representative experiment of two independent repetitions performed.

Two related regulatory proteins, PPP1R15A/GADD34 and PPP1R15B/CReP, encoded in mammals by *PPP1R15A* and *PPP1R15B*, direct the unspecific protein phosphatase 1 (PP1) to promote eIF2α^P^ dephosphorylation (4–7). PPP1R15A or PPP1R15B form a complex with PP1 via a conserved region of ∼70 amino acids (PPP1R15A residues 555–624) located at their C termini (5, 8–11) (Fig. 1*A*). This conserved C-terminal region of either PPP1R15 regulatory subunit is sufficient to promote eIF2α^P^ dephosphorylation and to inactivate the ISR (4, 5, 10, 11). Indeed, herpesviruses have exploited this activity and encode a small protein homologous to the C terminus of PPP1R15 to reverse eIF2α phosphorylation, undoing a defensive strategy of infected cells (12).

Despite genetic evidence pointing to the sufficiency of the conserved C-terminal portion of PPP1R15 in reversing the eIF2α^P^-dependent ISR *in vivo* (4, 5, 10), complexes formed *in vitro* between PPP1R15 regulatory subunit fragments and PP1 have not been observed to accelerate eIF2α^P^ dephosphorylation. Dephosphorylation of eIF2α^P^ is no faster by a complex of PPP1R15A–PP1 (or PPP1R15B–PP1) than by PP1 alone, showing that, when added as single components, PPP1R15A/B do not influence *k*_cat_ or *K_m_* of PP1 toward the substrate eIF2α^P^ (10). However, addition of G-actin to the binary complex of PPP1R15 and PP1 selectively accelerates eIF2α^P^ dephosphorylation. G-actin binds directly to the conserved C terminus of PPP1R15 alongside PP1 to form a ternary complex, whose affinity (*K_d_*∼10^−8^ m) matches the EC_50_ of G-actin's stimulatory effect (10, 13). The *in vivo* relevance of G-actin for eIF2α^P^ dephosphorylation is attested to by the finding that actin sequestration in fibers (as F-actin) enfeebles eIF2α^P^ dephosphorylation, implying a role for factors that affect the actin cytoskeleton in ISR regulation (14).

The ability to dephosphorylate eIF2α^P^ is an essential function in developing mammals (15). Nonetheless, inactivation of the *PPP1R15A* gene, which decelerates eIF2α^P^ dephosphorylation and prolongs the ISR, is protective in certain cellular and animal models of diseases associated with enhanced unfolded protein stress (16–19). This has generated interest in targeting the PPP1R15A-containing holophosphatase for inhibition by small molecules (reviewed in Ref. 20), an endeavor that requires detailed knowledge of the enzymatic mode of action.

A recent report challenged the need for G-actin as a co-factor in PPP1R15A-mediated eIF2α^P^ dephosphorylation (21). Instead, it suggested that a binary complex assembled from PP1α and a fragment of PPP1R15A (PPP1R15A^325–636^), encompassing both the C-terminal PP1-binding region and the N-terminal repeat–containing extension, dephosphorylates eIF2α^P^ faster than PP1 alone (21). Importantly, dephosphorylation of eIF2α^P^ by this active binary complex was reported to be selectively inhibited *in vitro* by guanabenz and Sephin1, two structurally related small molecules reputed to function *in vivo* as proteostasis modifiers (22, 23). The new study contradicts previous observations that neither a PPP1R15A–PP1 binary complex nor a PPP1R15A–PP1–G-actin ternary complex were susceptible to inhibition by guanabenz or Sephin1 (9, 13).

Here we address three important questions raised by these discrepant reports. Does the isotype of the PP1 catalytic subunit or its source (recombinant *versus* native) influence the requirement for G-actin by the eIF2α^P^-directed holophosphatase? What role does the N-terminal repeat–containing region of PPP1R15A play in eIF2α^P^ dephosphorylation by the holophosphatase? Do these factors influence the sensitivity of eIF2α^P^ dephosphorylation to guanabenz and Sephin1?

## Results

### Both native PP1 and bacterially expressed PP1α require the presence of G-actin to promote PPP1R15A-regulated eIF2α^P^ dephosphorylation

PP1 produced in *Escherichia coli* may differ in its enzymatic activity from PP1 purified from animal tissues, both in its substrate specificity and in its sensitivity to regulatory subunits (reviewed in Ref. 24). To determine whether the G-actin dependence of PP1–PPP1R15A–mediated eIF2α^P^ dephosphorylation is a peculiarity of the bacterially expressed PP1γ isoform used previously (10, 13), we purified the native catalytic subunit of PP1 from rabbit skeletal muscle (PP1^N^), following an established protocol (25), and compared the two PP1 preparations. Native PP1 (PP1^N^) is a mixture of PP1α, PP1β, and PP1γ isoforms and gave rise to two prominent bands on SDS-PAGE (Fig. S1*A*, *left panel*). The mass spectra of tryptic peptides derived from the PP1^N^ sample were analyzed by Maxquant with iBAQ (intensity-based absolute quant) to identify the major contaminating species (tropomyosin), and to estimate the relative contribution of PP1 and contaminants to the protein preparation. This enabled a comparison of the catalytic subunit content of PP1^N^ preparation with the bacterially expressed PP1γ, which served as a reference.

The N-terminal portion of PPP1R15A, which includes the membrane association region (26), compromises expression in bacteria and recovery of a functional protein (27). Therefore, we used a PPP1R15A^325–636^ fragment lacking this region, which is soluble when expressed in *E. coli*. Fig. 1*B* shows that addition of either PPP1R15A^325–636^-MBP (*lanes 5–8*) or G-actin alone (*lanes 13* and *14*) did not stimulate eIF2α^P^ dephosphorylation by nanomolar concentrations of PP1^N^. However, addition of both G-actin and PPP1R15A^325–636^-MBP (Fig. 1*B*, *lanes 9–12*) stimulated dephosphorylation by 5-fold, similar to the increase observed with bacterially expressed PP1γ (Fig. S1*B*) (10).

PP1 purified from rabbit muscle is a mixture of α, β, and γ isoforms, whereas it has been reported that the PP1α isoform possesses *in vivo* selectivity for PPP1R15A (6). Therefore, we prepared bacterially expressed PP1α by a method that promotes its native-like state (28). To control for effects the location of the tag might have on activity, we also generated an N-terminally MBP-tagged PPP1R15A^325–636^ (MBP-PPP1R15A^325–636^; Fig. 1*A* and Table S1). The holophosphatase comprised of PP1α and MBP-PPP1R15A^325–636^ also exhibited a stringent requirement for G-actin (Fig. 1*C*).

**Figure 2.**
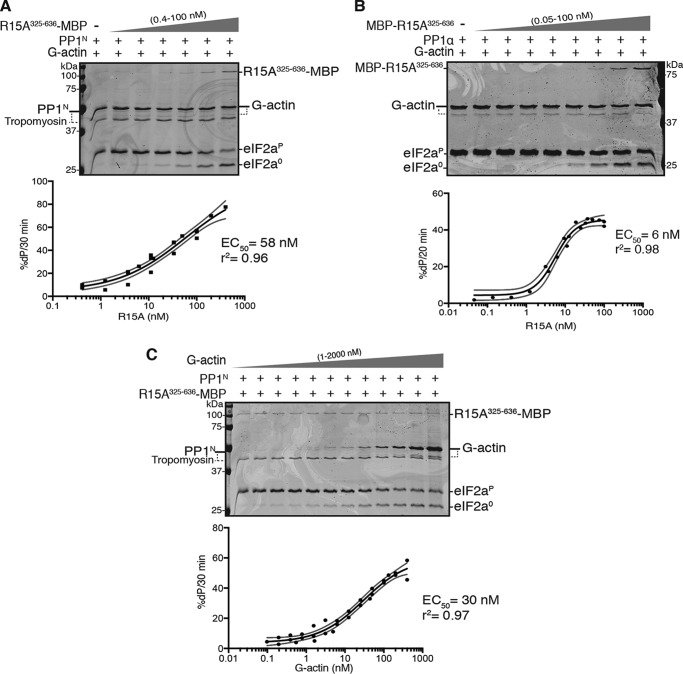
**The source of the catalytic subunit does not affect the kinetics of PPP1R15A and G-actin–mediated stimulation of eIF2α^P^ dephosphorylation.** *A*, *top panel*, Coomassie-stained PhosTag SDS-PAGE of dephosphorylation reactions (30 min at 30 °C) in which 2 μm eIF2α^P^ was dephosphorylated by PP1^N^ (20 nm) in the presence of G-actin (400 nm) and increasing concentrations of PPP1R15A^325–636^-MBP (0–100 nm). Shown is a representative experiment of three independent experiments performed. *Bottom panel*, plot of the rate of dephosphorylation of eIF2α^P^ as a function of PPP1R15A^325–636^-MBP concentration from the three experiments performed. The EC_50_ was calculated using the ”[Agonist] *versus* response − variable slope (four parameters)” function in GraphPad Prism v7. The *gray lines* represent the 95% confidence interval of the fitting. Shown are values obtained for EC_50_ and information of goodness of the fit (r^2^). *B*, as in *A* but using bacterially expressed PP1α (24 nm) and increasing concentrations of MBP-PPP1R15A^325–636^ (0–100 nm) in reactions performed over 20 min at 30 °C. Shown is a representative experiment of three independent experiments performed. *C*, as in *A* but with fixed concentrations of PP1^N^ (20 nm) and PPP1R15A^325–636^-MBP (50 nm) and varying the concentrations of G-actin (1–2000 nm). Shown is a representative experiment of three independent experiments performed.

A concentration-dependent stimulatory effect of PPP1R15A on eIF2α^P^ dephosphorylation by the three component holoenzyme (PP1, PP1R15A, and G-actin) was observed with constructs tagged at either their N or C termini and with either native or bacterially expressed PP1 (Fig. 2, *A* and *B*). The difference in EC_50_ values obtained for PPP1R15A^325–636^-MPB with PP1^N^ (58 nm) or MBP-PPP1R15A^325–636^ with PP1α (6 nm) may reflect the effect of the position of the MBP tag, the contaminating tropomyosin (in PP1^N^), or both. Importantly, the data agreed with similar experiments in which PPP1R15A^325–636^ and bacterially expressed PP1γ were used, with an EC_50_ of 10 nm (see Fig. 8*A* in Ref. 13).

G-actin also exerted a saturable concentration-dependent stimulatory effect on the activity of a three-component holophosphatase constituted with native PP1^N^ (Fig. 2*C*). The EC_50_ for G-actin with PP1^N^ (30 nm) was similar to that observed previously using bacterially expressed PP1γ, with an EC_50_ of 13 nm (see Fig. 2*C* in Ref. 13). Hence, despite variations in the estimated EC_50_ values for PPP1R15A or G-actin, the combinations of catalytic and regulatory subunits tested showed consistent PPP1R15A and G-actin concentration-dependent enzymatic activity. These experiments, conducted in a buffer of physiological ionic strength over a physiological protein concentration range (nanomolar catalytic subunit and micromolar substrate) and over a timescale aimed to minimize the effect of substrate depletion on enzyme kinetics, indicate that neither the source of PP1 nor the position of the tag in PPP1R15A are likely to account for the reported G-actin–independent ability of PPP1R15A to stimulate eIF2α^P^ dephosphorylation.

### Two-fold stimulation of eIF2α^P^ dephosphorylation by repeat-containing PPP1R15A in an unphysiological low ionic strength buffer

To explore the discrepant findings on the G-actin independent stimulatory activity of MBP-PPP1R15A^325–636^, we sought to reproduce the experiments reported in Ref. 21 as closely as possible. We received from the Bertolotti laboratory their expression plasmid. The encoded protein, referred to here as MBP∼PPP1R15A^325–636^ (Fig. 1*A*), differs from the one used above (MBP-PPP1R15A^325–636^) by the absence of three residues in the linker separating the MBP from PPP1R15A and 11 residues in the linker separating PPP1R15A from the C-terminal polyhistidine tag (Table S1). The MBP∼PPP1R15A^325–636^ fusion protein was produced in *E. coli* and purified as described previously (21), and dephosphorylation reactions were carried out in a salt-free, low ionic strength buffer designed to mimic as closely as possible the one used in that study (50 mm Tris (pH 7.4), 1.5 mm EGTA, and 2 mm MnCl_2_, with the notable exception of 0.5 mm TCEP, added here to prevent inactivation of the catalytic subunit by oxidation).

**Figure 3.**
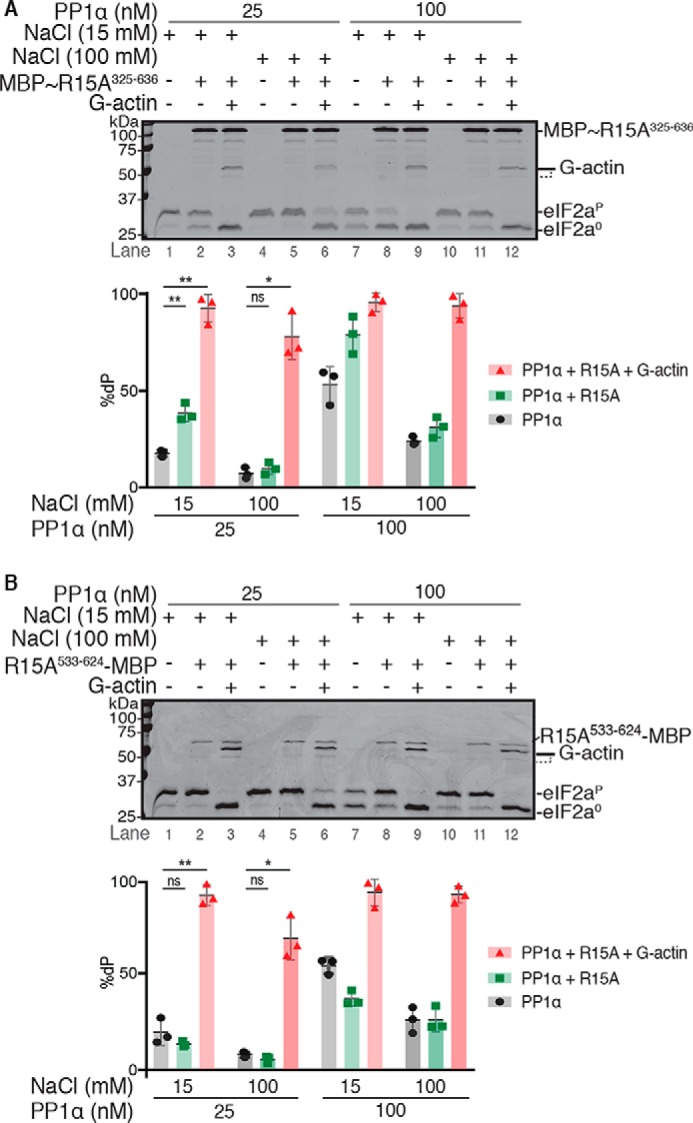
**PPP1R15A^325–636^ accelerates eIF2α^P^ dephosphorylation by PP1α in a low ionic strength buffer.** *A*, *top panel*, Coomassie-stained PhosTag SDS-PAGE containing resolved samples of dephosphorylation reactions (30 min at 30 °C) in which 2 μm eIF2αP was dephosphorylated by PP1α (25 or 100 nm) in the presence or absence of MBP∼PPP1R15A^325–636^ (1 μm) with or without G-actin (400 nm) in low (15 mm NaCl) or physiological (100 mm NaCl) ionic strength buffer. Shown is a representative experiment of three independent repetitions performed. *Bottom panel*, plot of the percentage of eIF2α^P^ dephosphorylation under the different conditions from the experiment above and the two other repeats performed. Statistical significance was derived from paired two-tailed *t* test (*ns*, nonsignificant, *p* > 0.05; *, *p* ≤ 0.05; **, *p* ≤ 0.01). *B*, as in *A* but using PPP1R15A^533–624^-MBP (200 nm). Shown is a representative experiment of three independent repetitions performed.

A 2-fold stimulation of eIF2α^P^ dephosphorylation by MBP∼PPP1R15A^325–636^ was apparent in reactions conducted at low salt concentration (15 mm) but lost at more physiological concentrations (100 mm), whereas the 5-fold stimulatory effect of G-actin was observed at both low and physiological salt concentration (Fig. 3*A*). The stimulatory effect of MBP∼PPP1R15A^325–636^ at low salt concentration depended on the N-terminal repeat–containing region of PPP1R15A (Fig. 3*B*), as reported previously (21), and was not observed with a nonspecific dephosphorylation substrate (Fig. S2*A*).

Although modest (2-fold) and confined to nonphysiological, low ionic strength conditions, this stimulatory effect was also reproducibly observed with the MBP-PPP1R15A^325–636^ and PPP1R15A^325–636^-MBP proteins used in Figs. 1 and 2 (Fig. S2*B*), negating a role for the linkers or the position of the tag in this activity. Notably, in both unphysiological low ionic strength buffer (in which PPP1R15A alone has a stimulatory effect) and under physiological conditions, the presence of G-actin dominates the kinetics of eIF2α^P^ dephosphorylation.

### Lengthy incubation of the enzymatic reactions does not uncover PPP1R15A's ability to promote G-actin–independent eIF2α^P^ dephosphorylation at physiological salt concentrations

**Figure 4.**
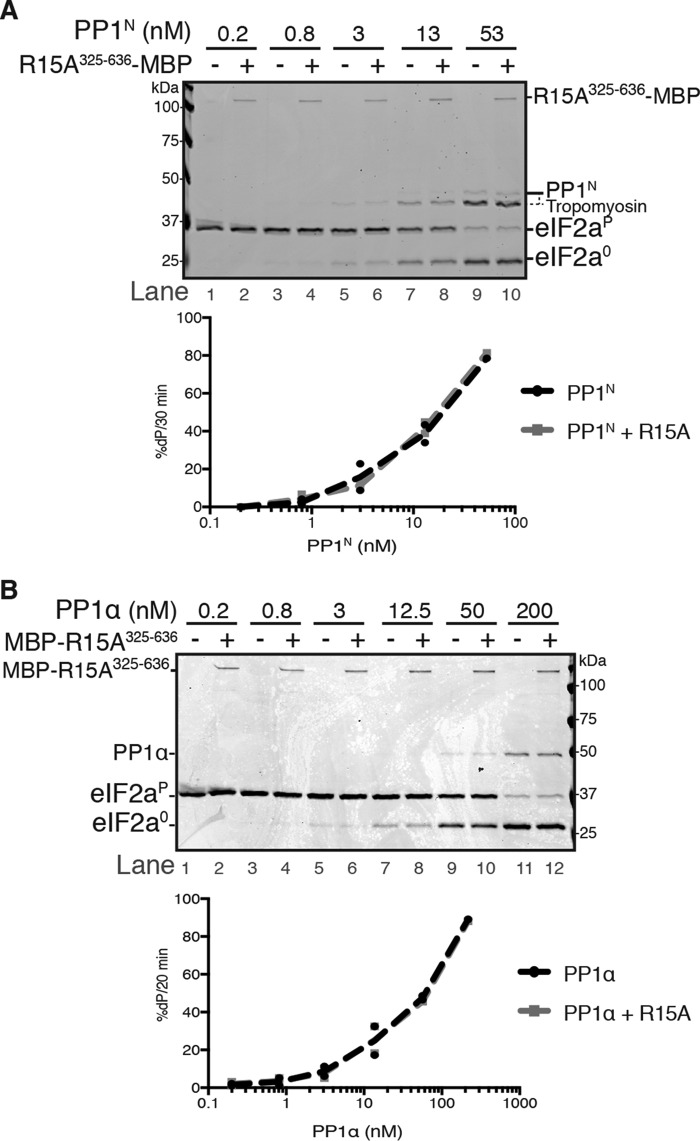
**At physiological ionic strength and in the absence of G-actin, PPP1R15A is unable to stimulate dephosphorylation of eIF2αP (despite extended incubation of 16 h).** *A*, *top panel*, Coomassie-stained PhosTag SDS-PAGE containing dephosphorylation reactions (16 h at 30 °C) in which 2 μm eIF2α^P^ was dephosphorylated by the indicated concentration of PP1^N^ in the presence or absence of PPP1R15A^325–636^-MBP (50 nm). Quantification of the percentage of dephosphorylation (%*dP*) is shown below the image. Shown is a representative experiment of two independent repetitions performed. *Bottom panel*, plot of the rate of dephosphorylation of eIF2α^P^ as a function of PP1^N^ concentration. Data were obtained by quantification of bands of images shown above and the other repeat performed. *B*, as in *A* but using PP1α as the source of the catalytic subunit and MBP-PPP1R15A^325–636^ (50 nm) as the regulatory subunit. Shown is a representative experiment of two independent repetitions performed.

Upon inhibition of the phosphorylating kinase, the eIF2α^P^ signal decays with a *t*_½_ of <10 min (with no change in the total eIF2α content) in both cultured mouse fibroblasts (see Fig. 6 in Ref. 14) and Chinese hamster ovary cells (see Fig. 10 in Ref. 13). Despite the rapid *in vivo* kinetics of the dephosphorylation reaction, the experiments pointing to G-actin–independent eIF2α^P^ dephosphorylation were conducted with long incubations of 16 h at 30 °C (21). In the absence of other components, PP1α is markedly unstable at 30 °C, losing about half of its activity by 1 h and all detectable activity by 3 h (Fig. S3, *A* and *B*). Thus, a stabilizing effect of a PP1 binding co-factor might have accounted for the apparent G-actin–independent stimulatory effect of MBP-PPP1R15A^325–636^ on PP1α-mediated eIF2α^P^ dephosphorylation at physiological salt concentrations. However, over a range of PP1 concentrations (0.2–200 nm), the presence of MBP-PPP1R15A^325–636^ failed to stimulate eIF2α^P^ dephosphorylation, regardless of whether PP1^N^ (Fig. 4*A*) or PP1α (Fig. 4*B*) was used as the catalytic subunit.

### Substrate recruitment by the repeat-containing PPP1R15A^325–512^ region plays a secondary role in the kinetics of eIF2α^P^ dephosphorylation, and its disruption is unlikely to account for sensitivity to Sephin1

**Figure 5.**
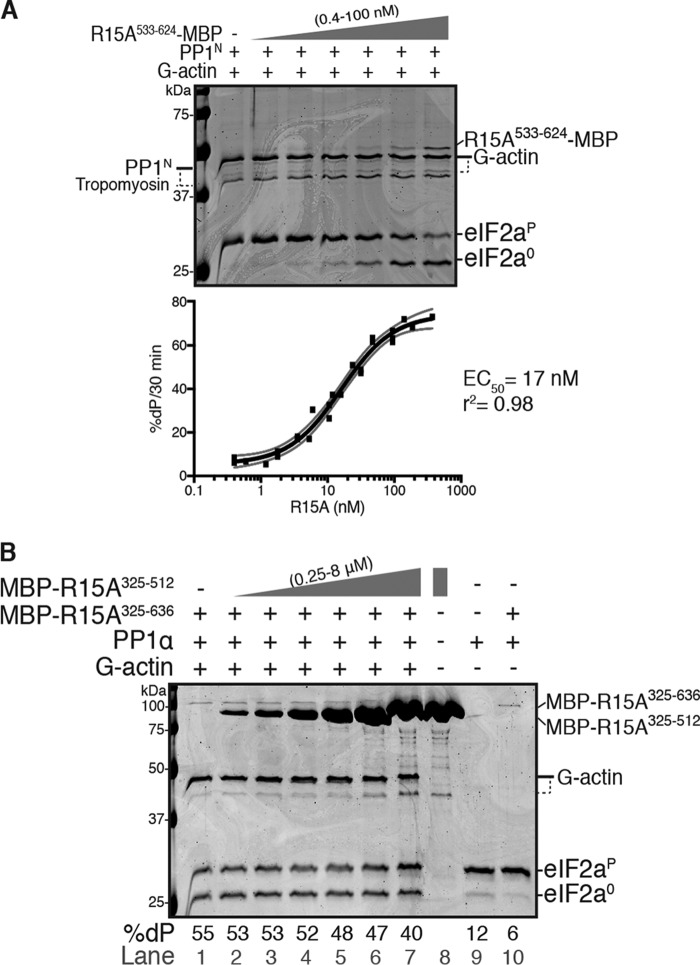
**The C-terminal portion of PPP1R15A is sufficient to promote eIF2α^P^ dephosphorylation.** *A*, *top panel*, Coomassie-stained PhosTag SDS-PAGE containing resolved samples from dephosphorylation reactions (30 min at 30 °C) in which 2 μm eIF2α^P^ was dephosphorylated by PP1^N^ (20 nm) in the presence of G-actin (400 nm) and increasing concentrations of PPP1R15A^533–624^-MBP (0–100 nm). Shown is a representative experiment of three independent repetitions performed. *Bottom panel*, plot of the rate of dephosphorylation of eIF2α^P^ as a function of PPP1R15A^533–624^-MBP concentration from the three experiments performed. The EC_50_ was calculated using the ”[Agonist] *versus* response − variable slope (four parameters)” function in GraphPad Prism v7. The *gray lines* represent the 95% confidence interval of the fitting. Shown are values obtained for EC_50_ and information of goodness of the fit (r^2^). *B*, as in *A* but using PP1α (24 nm) in the presence of MBP-PPP1R15A^325–636^ (24 nm), G-actin (400 nm), and increasing concentrations of MBP-PPP1R15A^325–512^ as a competitor (0–8 μm). The assays were performed during 20 min at 30 °C. *Lane 8*, loaded with only MBP-PPP1R15A^325–512^ shows the absence of a species co-migrating with eIF2α^0^ (which might otherwise obscure an inhibitory effect on dephosphorylation). *Lanes 9* and *10* control for the dependence of enzymatic activity on PPP1R15A and G-actin in this experiment. Quantification of the percentage of dephosphorylation (%*dP*) is shown below the image. Shown is a representative experiment of two independent repetitions performed.

PPP1R15A interacts directly with eIF2α, both in cells (9) and *in vitro* (21). This interaction maps to the repeat-containing region of PPP1R15A, residues 325–512, N-terminal to PPP1R15A's PP1-binding domain (Fig. 1*A*) and was proposed to play an important role in the catalytic cycle of PPP1R15A-containing holoenzymes (21). However, in the presence of G-actin, PPP1R15A^325–636^-MBP and PPP1R15A^533–624^-MBP (Fig. 1*A* and Table S1) stimulated eIF2α^P^ dephosphorylation similarly when paired either with PP1^N^ (compare our Figs. 2*B* and 5*A*) or with PP1γ (compare Figs. 8*A* and 2*B* in Ref. 13). These findings suggest that the conserved C-terminal PPP1R15 fragment that binds PP1 and G-actin simultaneously is sufficient to promote eIF2α^P^ dephosphorylation and to dominate its kinetics *in vitro* and call into question the importance of the N-terminal repeats in PPP1R15A to the fundamentals of the holoenzyme's catalytic cycle.

We considered that an important contributory role for substrate engagement by the PPP1R15A^325–533^ repeat-containing fragment to the catalytic cycle of the holophosphatase might have been masked by compensatory features that diverge between the different regulatory subunit constructs, fortuitously equalizing their activity. To address this possibility, we measured the ability of MBP-PPP1R15A^325–512^ containing the repeats but lacking the C-terminal PP1 binding region (Fig. 1*A* and Table S1) to compete with MBP-PPP1R15A^325–636^–mediated (G-actin–dependent) eIF2α^P^ dephosphorylation using PP1α as the catalytic subunit. Minimal inhibition of the dephosphorylation reaction was observed at competitor concentrations of up to 8 μm (Fig. 5*B*), which is a >300-fold excess over the MBP-PPP1R15A^325–636^ regulatory subunit (present in the reaction at 24 nm) and a concentration of 18-fold above the reported *K_d_* of the interaction between MBP-PPP1R15A^325–512^ and eIF2α^P^ (21).

**Figure 6.**
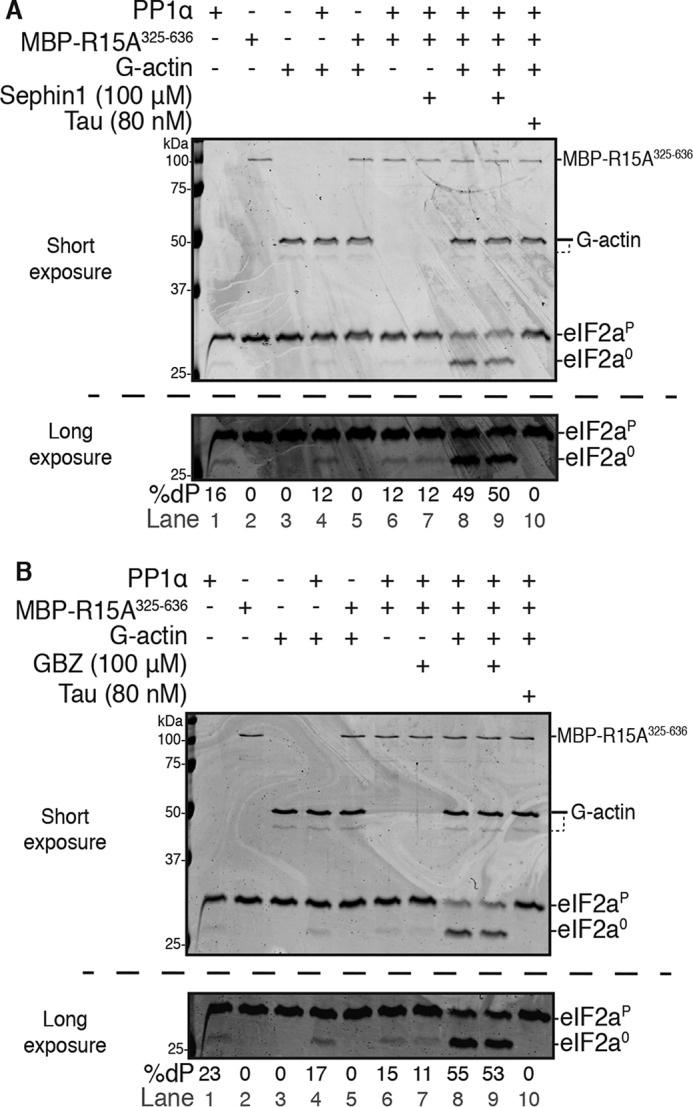
**Neither Sephin1 nor GBZ interfere with eIF2α^P^ dephosphorylation.** *A*, Coomassie-stained PhosTag SDS-PAGE containing resolved samples from dephosphorylation reactions (20 min, 30 °C) in which 2 μm eIF2α^P^ was dephosphorylated by PP1α (24 nm) in the presence or absence of MBP-PPP1R15A^325–636^ (60 nm) and/or G-actin (400 nm). The components were preincubated as specified with either Sephin1 (100 μm), tautomycin (80 nm), or DMSO (vehicle) for 15 min at room temperature before being added to the reaction. The *bottom panel* shows a long exposure of the relevant section of the image above corresponding to the phosphorylated and nonphosphorylated forms of eIF2α. Quantification of the percentage of dephosphorylation (%*dP*) is shown below the image. Shown is a representative experiment of three independent experiments performed. *B*, as in *A* but with guanabenz (*GBZ*). Shown is a representative experiment of two independent experiments performed.

These data suggest that substrate recruitment by the N-terminal extension of PPP1R15A plays a secondary role in the kinetics of the dephosphorylation reaction *in vitro* and that the reported role of Sephin1 and guanabenz in disrupting that interaction is unlikely to make an important contribution to their pharmacological activity. Consistent with these conclusions, we found that, under physiological salt conditions where eIF2α^P^ dephosphorylation depends on the concentration of PP1α, MBP-PPP1R15A^325–636^, and G-actin, we were unable to observe an inhibitory effect of either Sephin1 (Fig. 6*A* and Fig. S4*A*, *lanes 8–11*) or guanabenz (Fig. 6*B* and Fig. S4*B*, *lanes 8–11*) at a concentration of up to 100 μm, which exceeds by 100-fold the concentration required for a proteostatic effect in cultured cells (1 μm; see Fig. 1*F* in Ref. 23). Similarly, no effect of the compounds was observed on the PP1–PPP1R15A holophosphatase activity under low-salt conditions (Fig. S4, *A*, *lanes 1–7*, and *B*, *lanes 1–6*).

Complete inhibition of PPP1R15A-mediated eIF2α^P-^dephosphorylation by Sephin 1 was reported in an assay conducted over 16 h at 30 °C in a low ionic strength buffer (21). We wished to test whether the reported Sephin1 inhibition might be unmasked by this long incubation (in which the enzyme is undergoing inactivation; Fig. S3*B*). Using identical MBP∼PPP1R15A^325–636^ and PP1α constructs, in an identical low ionic strength buffer and following overnight incubation at 30 °C, we observed a 2-fold stimulation of eIF2α^P^ dephosphorylation by MBP∼PPP1R15A^325–636^ (similar to that noted in shorter reactions; Fig. 3). However, even under these conditions, designed to mimic as closely as possible those used in Ref. 21, the presence of 100 μm Sephin1 was devoid of an inhibitory effect on substrate dephosphorylation (Fig. S4*C*).

## Discussion

The new experiments presented here cover a range of conditions with realistic concentrations and time regimes. Incorporation of multiple time points and titrations of reaction components enabled a comparison of enzyme kinetics that accounts for the effect of substrate depletion. Our observations were made with four different PPP1R15A preparations, three different PP1 preparations, and both buffer conditions used previously in our laboratory and those used in Ref. 21, all of which consistently show the requirement for G-actin as an additional co-factor in enabling PPP1R15A to stimulate eIF2α^P^ dephosphorylation *in vitro*. Therefore, the results presented here are in keeping with previous observations that G-actin has an essential role in promoting eIF2α^P^ dephosphorylation both *in vitro* and *in vivo* (10, 13, 14).

The PP1 apo-enzyme is salt-sensitive and inhibited by buffers of physiological ionic strength (29). By contrast, PP1 holoenzymes retain their regulated enzymatic activity at physiological ionic strength (30). These considerations call into question the significance of the 2-fold stimulation of eIF2α^P^ dephosphorylation by PPP1R15A^325–636^ observed in buffer of low ionic strength. Our experiments also cast doubt on the importance of the physical interaction between the repeat-containing region of PPP1R15A (residues 325–512) and eIF2α^P^ in the substrate-specific dephosphorylation reaction carried out with physiological ionic strength. PPP1R15 regulatory subunits are found throughout the animal kingdom, but only their C-terminal ∼70 residues are conserved (11). This C-terminal fragment contains all the information needed to promote eIF2α^P^ dephosphorylation, as exemplified by its selective hijacking by herpesviruses (12) and by experimentally targeted expression in cells (see Fig. 1*C* in Ref. 10). In complex with G-actin, the conserved C-terminal fragment of the PPP1R15s is also able to direct PP1 to selectively dephosphorylate eIF2α^P^ *in vitro* (Figs. 2 and 5*A* here and Refs. 10, 13).

**Figure 7.**
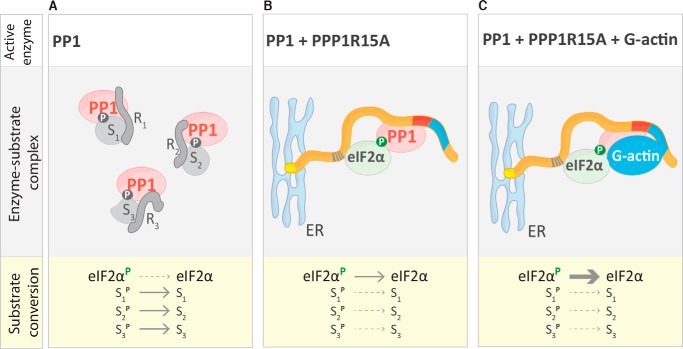
**Model depicting PPP1R15A's role in regulating eIF2α^P^ dephosphorylation.** *A*, in the absence of PPP1R15A, the cellular pool of the catalytic subunit (*PP1*) is preferentially bound by a variety of regulatory subunits (*R1*, *R2*, and *R3*), which direct its phosphatase activity toward their specific substrates (*S1*, *S2*, and *S3*), excluding eIF2α^P^. In the *Substrate conversion* section, see the preferential dephosphorylation of substrates S1, S2, and S3 (*solid arrows*) compared with eIF2α (*dotted arrows*). *B*, rising levels of PPP1R15A recruit PP1 away from other regulatory subunits, redirecting its phosphatase activity toward eIF2α^P^ by excluding other substrates. In the *Substrate conversion* section, observe the inverted preferential dephosphorylation of substrates compared with *A. C*, when present, G-actin joins the PPP1R15A–PP1 holophosphatase, increasing its intrinsic eIF2α^P^-directed catalytic activity. In the *Substrate conversion* section, see the increased *arrow thickness* for eIF2α^P^ dephosphorylation compared with *B*.

The prominent stimulatory role of G-actin on eIF2α^P^ dephosphorylation, observed both *in vivo* and *in vitro*, should not obscure the possibility that binary complex formation with PPP1R15 might also favor eIF2α^P^ dephosphorylation independently of G-actin joining the complex. Regulatory subunit binding restricts access to PP1 (24, 31), favoring the phosphorylation of one class of substrates over another. Mere exclusion of some substrates from access to the catalytic subunit might accelerate eIF2α^P^ dephosphorylation when levels of PPP1R15A levels are sufficiently elevated in cells, even though *in vitro* (and in the absence of competing substrates), the PPP1R15A-PP1 binary complex is not a faster eIF2α^P^ phosphatase than PP1 alone (provided the experiments are conducted at physiological salt concentrations). As neither Sephin1 nor guanabenz affect the stability of the PPP1R15A–PP1 complex (13), it is unlikely that they achieve any measure of inhibition by weakening PPP1R15A's ability to compete with other regulatory subunits for limiting amounts of catalytic subunit. These considerations lead us to propose a dual role for PPP1R15A in cells: diverting limiting amounts of PP1 away from other substrates toward eIF2α^P^ and, in conjunction with G-actin as an essential co-activator, stimulating the intrinsic rate of dephosphorylation by the holoenzyme thus formed. Actin, too, has a dual role in stimulating eIF2α^P^ dephosphorylation: by stabilizing the PPP1R15-PP1 complex (14), G-actin favors the exclusion of other regulatory subunits while stimulating enzyme kinetics selectively toward eIF2α^P^ (Fig. 7).

Here we present no argument against an important function for the divergent N-terminal extensions of PPP1R15 regulatory subunits. This role may play out in terms of subcellular localization (26) or protein stability (32) and might be influenced by a physical interaction with the substrate (9, 21). However, our findings argue that the physical interaction noted previously between PPP1R15A residues 325–512 and eIF2α^P^ (21) is unlikely to play an important role in formation of the enzyme–substrate complex required for catalysis under physiological conditions, and, hence, its reported disruption by guanabenz or Sephin1 is unlikely to underscore an inhibitory effect on eIF2α^P^ dephosphorylation.

Most importantly perhaps, the findings presented here argue that the inability of previous efforts to uncover a role for guanabenz or Sephin1 in inhibiting eIF2α^P^ dephosphorylation *in vitro* (9, 13) was unlikely to have arisen from choice of catalytic subunit, from features of the PPP1R15A regulatory subunit, or the buffer conditions used. Rather, the findings reported here, made *in vitro*, reinforce observations that Sephin1 and guanabenz have no measurable effect on the rate of eIF2α^P^ dephosphorylation in cells (13). The recent description of PPP1R15A/GADD34-independent cellular effects of guanabenz (33) and our observations that Sephin1-induced changes in gene expression were noted both in cells lacking PPP1R15A and in cells with nonphosphorylatable eIF2α (13) suggest the need to reconsider the role of these two compounds as eIF2α^P^ dephosphorylation inhibitors.

## Experimental procedures

### Protein expression and purification

The plasmids used to express protein in *E. coli* and the sequence of the encoded proteins are listed in Tables S1 and S2.

PPP1R15A^325–636^-MBP and PPP1R15A^533–624^-MBP were produced as described previously (13). Briefly, proteins were expressed in *E. coli* BL21 (New England Biolabs, catalog no. C3013) as N-terminally tagged GSH *S*-transferase fusion proteins and purified by tandem affinity chromatography, bound to a GSH-Sepharose 4B resin and eluted with GSH, followed by an overnight cleavage with tobacco etch virus protease (to remove the glutathione *S*-transferase tag), binding to amylose beads, and elution in maltose-containing buffer.

MBP-PPP1R15A^325–636^ and MBP-PPP1R15A^325–512^ were constructed in the C-terminally hexahistidine tag–containing pMAL-c5x-His plasmid (New England Biolabs, catalog no. N8114). Transformed *E. coli* BL21 (New England Biolabs, catalog no. C3013) were selected on lysogeny broth (LB) agar plates supplemented with 100 μg/ml ampicillin. A single colony was picked to grow overnight in 5 ml of starter culture that served to inoculate 2 liters of lysogeny broth (LB) (all supplemented with 100 μg/ml ampicillin), which was kept at 37 °C. At *A*_600_ = 0.6–0.8, protein expression was induced using 1 mm isopropyl β-d-thiogalactopyranoside at 18 °C for 20 h. Bacteria were pelleted and resuspended in ice-cold His_6_ lysis buffer containing 50 mm Tris (pH 7.4), 500 mm NaCl, 1 mm MgCl_2_, 1 mm tris(2-carboxyethyl)phosphine (TCEP), 100 μm phenylmethylsulfonyl fluoride, 20 trypsin inhibitory units per liter aprotinin, 2 μm leupeptin, 2 μg/ml pepstatin, 20 mm imidazole, and 10% glycerol. Bacterial suspensions were lysed using an Emulsi-Flex-C3 homogenizer (Avestin, Inc., Ottawa, ON, Canada) and clarified in a JA-25.50 rotor (Beckman Coulter) at 33,000 × *g* for 30 min at 4 °C. Pre-equilibrated nickel-nitrilotriacetic acid beads (Qiagen, catalog no. 30230) were incubated with the samples for 2 h at 4 °C. Proteins were eluted in 2 ml of imidazole elution buffer (50 mm Tris (pH 8), 100 mm NaCl, 500 mm imidazole, and 10% glycerol) and incubated with amylose beads (New England Biolabs, catalog no. E8021S) pre-equilibrated with lysis buffer (His_6_ lysis buffer without imidazole) for 2 h at 4 °C. The amylose beads were batch-washed using 25 bed volumes of lysis buffer, and proteins were eluted with amylose elution buffer (lysis buffer + 10 mm maltose). MBP-R15A^325–512^ purification required an additional buffer exchange step (into lysis buffer) using Centripure P1 desalting columns (EMP Biotech, catalog no. CP-0110) to eliminate maltose (which appeared to interfere with the dephosphorylation reactions when present at high concentrations).

MBP∼PPP1R15A^325–636^ (a gift from the Bertolotti laboratory) was expressed and purified as described previously (21) with minor modifications. The isopropyl β-d-thiogalactopyranoside–induced culture was maintained for 16 h at 18 °C, and 0.5 mm TCEP was included in all buffers, throughout the purification procedure, and in the final dialysis buffer (50 mm Tris (pH 7.4), 200 mm NaCl, and 0.5 mm TCEP).

For eIF2α^P^, the N-terminal fragment of human eIF2α (1–185, with three solubilizing mutations) was purified from bacteria and phosphorylated *in vitro* using the kinase domain of PKR-like ER kinase (PERK), as described previously (10). G-actin was purified from rabbit muscle according to Ref. 34 as modified in Ref. 10. PP1γ (7–300) was purified according to Ref. 13. PP1α (7–330) was purified from BL21 *E. coli* according to Refs. 28, 35. PP1^N^ was purified from rabbit muscle according to Ref. 25.

### In vitro dephosphorylation reactions

Unless otherwise stated, dephosphorylation reactions were performed at a final volume of 20 μl by assembling 5 μl of 4× solution of each component: PP1, PPP1R15A, G-actin, and eIF2α^P^ (or their respective buffers). A 10× assay buffer (500 mm Tris (pH 7.4), 1 m NaCl, 1 mm EDTA, 0.1% Triton X-100, and 10 mm MgCl_2_) was diluted 1:10, supplemented with 1 mm DTT, and used to create working solutions of PP1, PPP1R15A, and eIF2α^P^ at the desired concentrations. G-actin working solutions were created using G buffer (2 mm Tris-HCl (pH 8), 0.2 mm ATP, 0.5 mm DTT, and 0.1 mm CaCl_2_). Holoenzyme components (PP1, PPP1R15A, and G-actin) were combined first, and substrate (eIF2α^P^) was added last to initiate the reactions, which were conducted under shaking at 500 rpm and at 30 °C for the specified time. The final buffer composition was 36 mm Tris (pH 7.4), 76 mm NaCl, 74 μm EDTA, 0.007% Triton X-100, 0.7 mm MgCl_2_, 25 μm CaCl_2_, 0.05 mm ATP, 0.8 mm DTT, 0.5 μm Latrunculin B, 0.4–3 μm MnCl_2_, 0.5% glycerol, and 50 μm TCEP in the experiments performed for Figs. 1, 2, and 4–6 and Figs. S1, S3, and S4, *A*, *lanes 8–11*, and *B*, *lanes 7–10*.

Dephosphorylation reactions designed to reproduce the observations in Ref. 21 were performed in the assay buffer described therein (50 mm Tris-HCl (pH 7.4), 1.5 mm EGTA (pH 8.0), and 2 mm MnCl_2_), with the modification that 0.5 mm TCEP was added to disfavor oxidative inactivation of the enzyme. The NaCl content of the final reaction was constrained by the contribution of the protein solutions added to each reaction. To maintain parity between reactions performed with and without PPP1R15A, an equal volume of the PPP1R15A buffer was added to reactions lacking the protein. The final salt concentration in the various reactions is noted in the figure legends.

The stability test of PP1α (Fig. S3) was performed by preparing a fresh 240 nm solution of PP1α in the assay buffer described above. Separate aliquots were preincubated either at 30 °C or on ice for the specified times (30 min to 7 h, see schematic in Fig. S3*A*). At termination of the preincubation, 5 μl of these preincubated solutions were added to 20 μl of dephosphorylation reactions as described above.

Dephosphorylation reactions to test the activity of Sephin1 or guanabenz (Fig. 6 and Fig. S4) included 15-min preincubation of the enzymatic components at room temperature (before the addition of substrate) with either Sephin1 (Enamine, catalog no. EN300-195090), guanabenz (Sigma-Aldrich, catalog no. D6270), tautomycin (Calbiochem, catalog no. 5805551), or an equal volume of DMSO (vehicle).

Reactions were terminated by addition of 10 μl of 3× Laemmli buffer supplemented with 100 mm DTT and heating the samples for 5 min at 70 °C. A third (10 μl) of the final volume was resolved in 12.5% PhosTag SDS gels (Wako, catalog no. NARD AAL-107) at 200 V for 1 h. Gels were stained with Coomassie Instant Blue and imaged on an Odyssey imager (LI-COR, Lincoln, NE).

ImageJ was used to quantify eIF2α^P^ dephosphorylation, as reflected by the intensity of the fluorescence arising from the Coomassie stain of the eIF2α^P^ and eIF2α^0^ bands resolved by the PhosTag SDS-PAGE gels and captured as a TIF file on the Odyssey imager. GraphPad Prism v8 was used to fit the plot and perform statistical analysis. Table S3 lists the number of times each experiment was performed.

## Author contributions

A. C.-C. and D. R. conceptualization; A. C.-C. data curation; A. C.-C. formal analysis; A. C.-C. investigation; A. C.-C., Z. C., M. S. C., W. P., M. B., and D. R. writing-review and editing; D. R. supervision; D. R. funding acquisition; D. R. writing-original draft; D. R. project administration. A. C.-C. conceived the study, co-designed and conducted the experiments, interpreted the results, created the figures, and co-wrote the paper. Z. C. co-designed the experiments, assisted with the preparation of PP1 from rabbit muscle, interpreted the results, and edited the manuscript. M. S. C. expressed and purified PP1α from *E. coli*, interpreted the results, and edited the manuscript. W. P. oversaw the expression and purification of PP1α from *E. coli*, interpreted the results, and edited the manuscript. M. B. co-designed the experiments, oversaw the purification of PP1 from rabbit muscle, interpreted the results, and edited the manuscript. D. R. conceived the study, co-designed the experiments, interpreted the results, and co-wrote the paper.

## Supplementary Material

Supporting Information

## Footnotes

6 The abbreviations used are:

ISR: integrated stress response
eIF: eukaryotic initiation factor
MBP: maltose-binding protein
ER: endoplasmic reticulum
TCEP: tris(2-carboxyethyl)phosphine.
