# Supplementary material for: A Sephin1-insensitive tripartite holophosphatase dephosphorylates translation initiation factor 2α
Source: J Biol Chem. 2018 Apr 4;293(20):7766–76. doi: 10.1074/jbc.RA118.002325 (PMC5961032; doi:10.1074/jbc.RA118.002325)
Supplement: Supporting Information [file supp_293_20_7766__index.html]

A Sephin1-insensitive tripartite holophosphatase dephosphorylates translation initiation factor 2α — Reconstitution of a PPP1R15A-containing holophosphatase — A Sephin1-insensitive tripartite holophosphatase dephosphorylates translation initiation factor 2α — Reconstitution of a PPP1R15A-containing holophosphatase — Supporting Information 

# A Sephin1-insensitive tripartite holophosphatase dephosphorylates translation initiation factor 2α

## Supporting Information

- Supporting Information \_Figures & Tables - Supporting Information \_Figures & Tables
