# Supplementary material for: A Sephin1-insensitive tripartite holophosphatase dephosphorylates translation initiation factor 2α
Source: J Biol Chem. 2018 Apr 4;293(20):7766–76. doi: 10.1074/jbc.RA118.002325 (PMC5961032; doi:10.1074/jbc.RA118.002325)
Supplement: Supporting Information [file supp_RA118.002325_135881_1_supp_111201_p6nrr3.pdf]

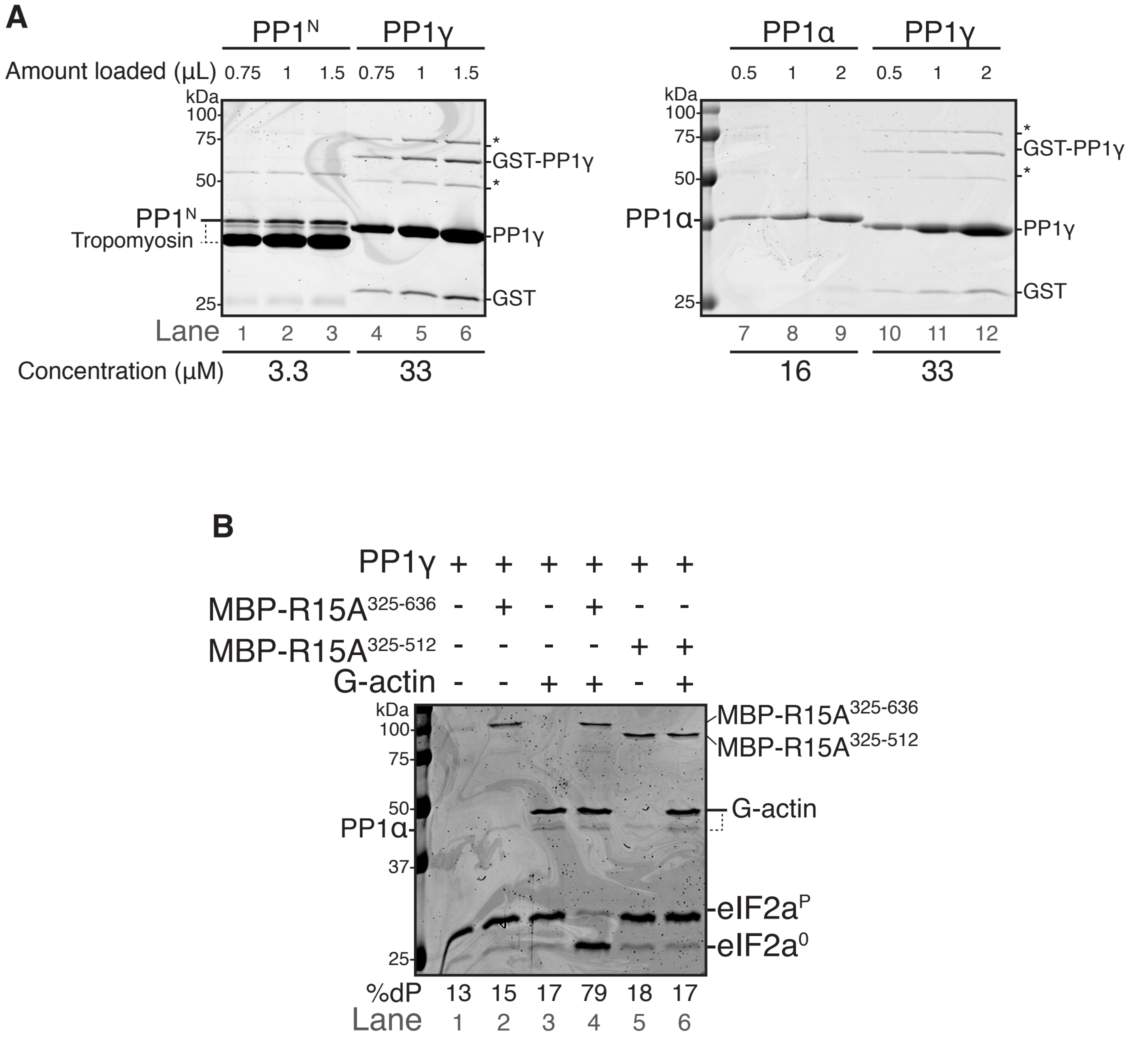

Supporting Figure 1. Analysis of the purity of the different sources of PP1. *A*, Coomassie-stained SDS-PAGE in which different amounts of PP1 sample have been resolved. The PP1^{N} preparation gave rise to two bands: a PP1 and tropomyosin band. The PP1γ preparation contained some free Glutathione S Transferase (GST) and GST-PP1 fusion protein from the purification steps, as well as other minor contaminants (*). The PP1 concentration in the different preparations is shown below the panels, calculated using PP1γ as a reference. *B*, Coomassie-stained PhosTag-SDS-PAGE containing resolved samples from dephosphorylation reactions (as in Fig. 1*A* and *B*) in which 2 μM eIF2α^{P} was dephosphorylated using bacterially-expressed PP1γ (24 nM) in presence or absence of MBP-PPP1R15A$^{325\text{-}636}$ (50 nM), MBP-PPP1R15A$^{325\text{-}512}$ (50 nM) and/or G-actin (400 nM) for 20 minutes at 30˚C. Quantification of percentage of dephosphorylation (%dP) is shown below the image.

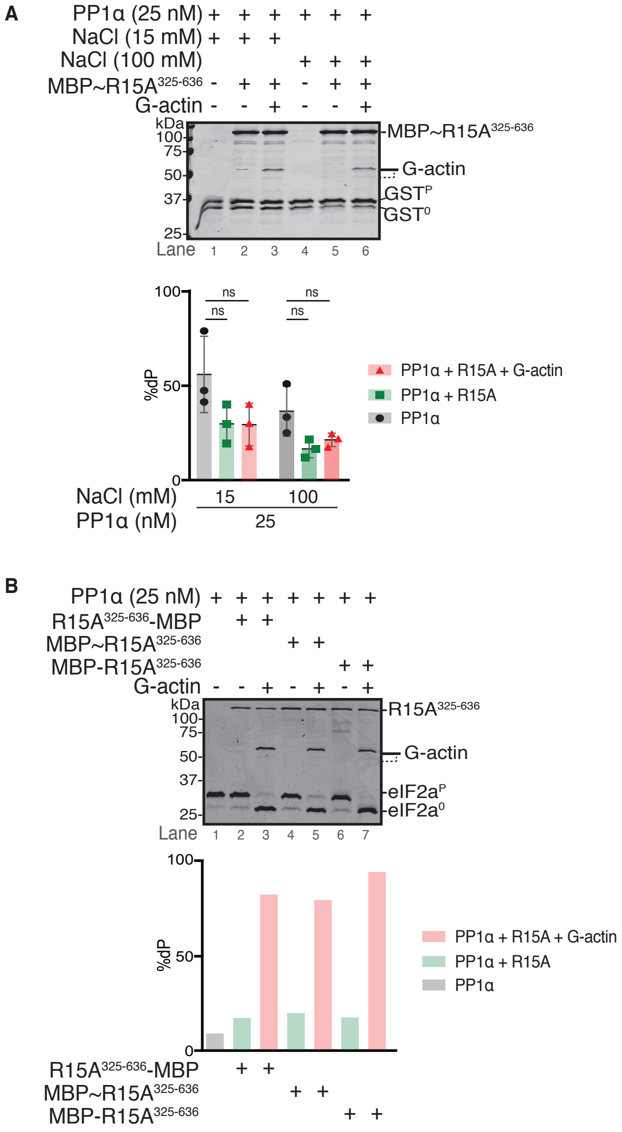

Supporting Figure 2. PPP1R15A$^{325-636}$ selectively accelerates eIF2α^{P} dephosphorylation by PP1α in a low ionic strength buffer. *A*, Upper panel: Coomassie-stained PhosTag-SDS-PAGE containing resolved samples of dephosphorylation reactions (30 minutes at 30˚C) in which 2 µM GST^{P} (a non specific substrate) was dephosphorylated by PP1α (25nM) in the presence or absence of MBP~PPP1R15A$^{325-636}$ (1 µM) with or without G-actin (400 nM) in low (15 mM NaCl) ionic strength buffer. Shown is a representative experiment of three independent repetitions performed. Lower panel: Plot of the percentage of eIF2α^{P} dephosphorylation at the different conditions from the experiment above and the two other repeats performed. Statistical significance derived from paired two tailed t-test,(ns, non significant, $p > 0.05$). *B*, as in "*A*" but using PPP1R15A$^{325-636}$-MBP (200 nM), MBP~PPP1R15A$^{325-636}$ (200 nM) or MBP-PPP1R15A$^{325-636}$ (200 nM) as regulatory subunits with and without G-actin (400 nM) in low (15 mM NaCl) ionic strength buffer.

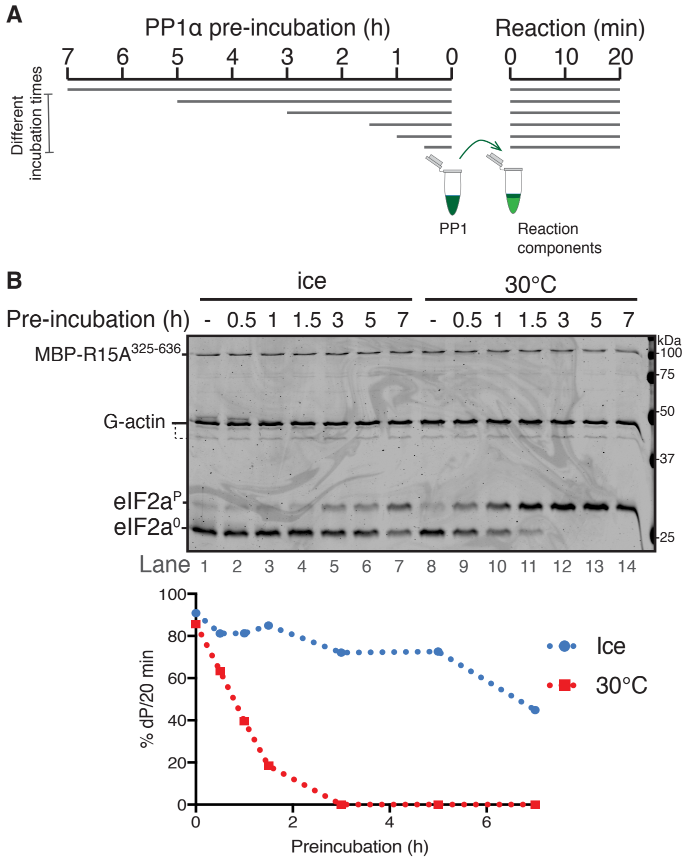

Supporting Figure 3. PP1α is an unstable enzyme. *A*, Schema of the experiment. Samples of PP1α (at 240 nM) were pre-incubated for the indicated period of time, either on ice or at 30˚C, before being diluted into a eIF2α^{P} dephosphorylation reaction. *B*, Upper panel: Coomassie-stained PhosTag-SDS-PAGE containing samples from dephosphorylation reactions (20 minutes at 30˚C) in which 2 μM eIF2α^{P} was dephosphorylated by the pre-incubated PP1α (60 nM) in presence of MBP-PPP1R15A$^{325\text{-}636}$ (60 nM) and G-actin (400 nM). Lower panel. Plot of the rate of dephosphorylation of eIF2α^{P} as a function of pre-incubation time of PP1α catalytic subunit. Data was obtained by quantification of bands of image shown above.

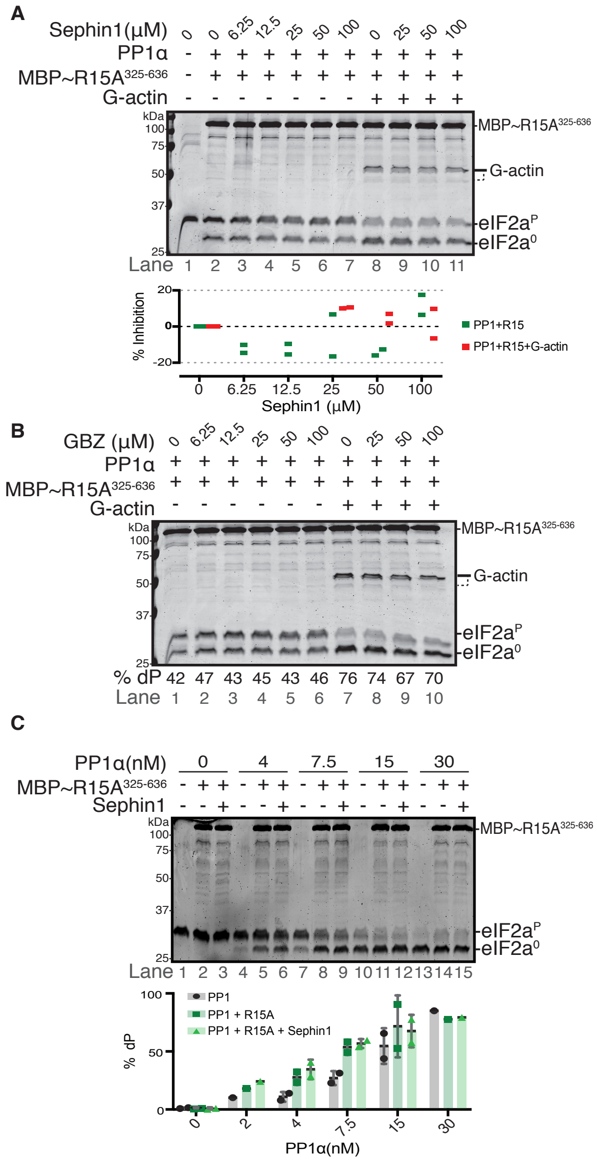

Supporting Figure 4. Neither Sephin1 nor GBZ interfere with eIF2α^{P} dephosphorylation. *A*, Upper panel: Coomassie-stained PhosTag-SDS-PAGE containing resolved samples from dephosphorylation reactions (30 minutes, 30˚C) in which 2 µM eIF2α^{P} were dephosphorylated by PP1α (25 nM) in presence of MBP~PPP1R15A$^{325-636}$ (1 µM) in either low ionic strength buffer (15 mM NaCl, without G-actin, lanes 2-7), or in physiological ionic strength buffer (100 mM, with 400 nM G-actin, lanes 8-11) in presence of Sephin1 (or DMSO carrier). Shown is a representative of two independent experiments performed. Lower panel: Plot of percentage inhibition observed in all samples of the experiment shown above and its repeat (calculated separately in each experiment relative to the no-compound condition, 0 µM). *B*, as in "A" but with Guanabenz (GBZ). Percentage of dephosphorylation shown below the image. *C*, Upper panel: as in "*A*" but following extended incubation time of dephosphorylation reactions (18 hour, 30˚C) performed in presence of the indicated concentrations of PP1α in the absence or presence of MBP~PPP1R15A$^{325-636}$ (1 µM) and Sephin1 (100 µM). Shown is a representative of two independent experiments performed. Lower panel: Plot of the percentage of eIF2α^{P} dephosphorylation from the experiment above and the other repeat performed

Table S1. PPP1R15A protein variants used in this study

Name	Sequence	Encoded in plasmid (See Table S2)
**PPP1R15A(325-636)-MBP**	SGLNDIFEAQKIEWHEGSDPEAECPPCIPPPSAFLKAWVYWPGEDTEEEEDEEEDEDSDSGSDEEEGEAEASSSTPATGVFLKSWVYQPGEDTEEEEDEDSDTGSAEDEREAETSASTPPASAFLKAWVYRP<br>GEDTEEEEDEDVDSEDKEDDSEAALGEAESDPHPSHPDQRAHFRGWGYRPGKETEEEEAAEDWGEAEPCPFRVAIYVPGEKPPPPWAPPRLPLRLQRRLKRPETPTHDPDPETPLKARKVRFSEKVTVHFL<br>AVWAGPAQAARQGPWEQLARDRSRFARRIAQAQEELSPCLTPAARARAWARLRNPPLAPIPALTQTLPSLQAKIEEGKLVIWINGDKGYNGLAEVGKKFEKDTGIKVTVEHPDKLEEKFPQVAATGDGPDII<br>FWAHDRFGGYAQSGLLAEITPDKAFQDKLYPFTWDAVRYNGKLIAYPIAVEALSLIYNKDLLPNPPKTWEEIPALDKELKAKGKSALMFNLQEPYFTWPLIAADGGYAFKYENGKYDIKDVGVDNAGAKA<br>GLTFLVDLIKNKHMNADTDYSIAEAAFNKGETAMTINGPWAWSNIDTSKVNYGVTVLPTFKGQPSKPFVGVLSAGINAASPNKELAKEFLENYLLTDEGLEAVNKDKPLGAVALKSYEEELAKDPRIAAT<br>MENAQKGEIMPNIPQMSAFWYAVRTAVINAASGRQTVDEALKDAQTRITK*	UK1921
**PPP1R15A(533-624)MBP**	SGLNDIFEAQKIEWHEGSDPGRRLKRPETPTHDPDPETPLKARKVRFSEKVTVHFLAVWAGPAQAARQGPWEQLARDRSRFARRITQAQEELSPCLTPAARARAWARLRNPPLQAKIEEGKLVIWINGDKG<br>YNGLAEVGKKFEKDTGIKVTVEHPDKLEEKFPQVAATGDGPDIIFWAHDRFGGYAQSGLLAEITPDKAFQDKLYPFTWDAVRYNGKLIAYPIAVEALSLIYNKDLLPNPPKTWEEIPALDKELKAKGKSAL<br>MFNLQEPYFTWPLIAADGGYAFKYENGKYDIKDVGVDNAGAKAGLTFLVDLIKNKHMNADTDYSIAEAAFNKGETAMTINGPWAWSNIDTSKVNYGVTVLPTFKGQPSKPFVGVLSAGINAASPNKEL<br>AKEFLENYLLTDEGLEAVNKDKPLGAVALKSYEEELAKDPRIAATMENAQKGEIMPNIPQMSAFWYAVRTAVINAASGRQTVDEALKDAQTRITK*	UK1920
**MBP-PPP1R15A(325-512)**	MKIEEGKLVIWINGDKGYNGLAEVGKKFEKDTGIKVTVEHPDKLEEKFPQVAATGDGPDIIFWAHDRFGGYAQSGLLAEITPDKAFQDKLYPFTWDAVRYNGKLIAYPIAVEALSLIYNKDLLPNPPKTWE<br>EIPALDKELKAKGKSALMFNLQEPYFTWPLIAADGGYAFKYENGKYDIKDVGVDNAGAKAGLTFLVDLIKNKHMNADTDYSIAEAAFNKGETAMTINGPWAWSNIDTSKVNYGVTVLPTFKGQPSKPFV<br>GVLSAGINAASPNKELAKEFLENYLLTDEGLEAVNKDKPLGAVALKSYEEELVKDPRIAATMENAQKGEIMPNIPQMSAFWYAVRTAVINAASGRQTVDEALKDAQTNSSSNNNNNNNNNNLGIEGRISH<br>MSMGGREAECPPCIPPPSAFLKAWVYWPGEDTEEEEDEEEDEDSDSGSDEEEGEAEASSSTPATGVFLKSWVYQPGEDTEEEEDEDSDTGSAEDEREAETSASTPPASAFLKAWVYRPGEDTEEEEDEDVD<br>SEDKEDDSEAALGEAESDPHPSHPDQRAHFRGWGYRPGKETEEEEAAEDWGEAEPCPFRVAIYVDGSEFPAGNHHHHHH*	UK2261
**MBP~PPP1R15A(325-636)**	MKIEEGKLVIWINGDKGYNGLAEVGKKFEKDTGIKVTVEHPDKLEEKFPQVAATGDGPDIIFWAHDRFGGYAQSGLLAEITPDKAFQDKLYPFTWDAVRYNGKLIAYPIAVEALSLIYNKDLLPNPPKTWE<br>EIPALDKELKAKGKSALMFNLQEPYFTWPLIAADGGYAFKYENGKYDIKDVGVDNAGAKAGLTFLVDLIKNKHMNADTDYSIAEAAFNKGETAMTINGPWAWSNIDTSKVNYGVTVLPTFKGQPSKPFV<br>GVLSAGINAASPNKELAKEFLENYLLTDEGLEAVNKDKPLGAVALKSYEEELVKDPRIAATMENAQKGEIMPNIPQMSAFWYAVRTAVINAASGRQTVDEALKDAQTNSSSNNNNNNNNNNLGIEGRISH<br>MSMEAECPPCIPPPSAFLKAWVYWPGEDTEEEEDEEEDEDSDSGSDEEEGEAEASSSTPATGVFLKSWVYQPGEDTEEEEDEDSDTGSAEDEREAETSASTPPASAFLKAWVYRPGEDTEEEEDEDVDSED<br>KEDDSEAALGEAESDPHPSHPDQRAHFRGWGYRPGKETEEEEAAEDWGEAEPCPFRVAIYVPGEKPPPPWAPPRLPLRLQRRLKRPETPTHDPDPETPLKARKVRFSEKVTVHFLAVWAGPAQAARQGPW<br>EQLARDRSRFARRITQAQEELSPCLTPAARARAWARLRNPPLAPIPALTQTLPSHHHHHHSG*	UK2258
**MBP-PPP1R15A(325-636)**	MKIEEGKLVIWINGDKGYNGLAEVGKKFEKDTGIKVTVEHPDKLEEKFPQVAATGDGPDIIFWAHDRFGGYAQSGLLAEITPDKAFQDKLYPFTWDAVRYNGKLIAYPIAVEALSLIYNKDLLPNPPKTWE<br>EIPALDKELKAKGKSALMFNLQEPYFTWPLIAADGGYAFKYENGKYDIKDVGVDNAGAKAGLTFLVDLIKNKHMNADTDYSIAEAAFNKGETAMTINGPWAWSNIDTSKVNYGVTVLPTFKGQPSKPFV<br>GVLSAGINAASPNKELAKEFLENYLLTDEGLEAVNKDKPLGAVALKSYEEELVKDPRIAATMENAQKGEIMPNIPQMSAFWYAVRTAVINAASGRQTVDEALKDAQTNSSSNNNNNNNNNNLGIEGRISH<br>MSMGGREAECPPCIPPPSAFLKAWVYWPGEDTEEEEDEEEDEDSDSGSDEEEGEAEASSSTPATGVFLKSWVYQPGEDTEEEEDEDSDTGSAEDEREAETSASTPPASAFLKAWVYRPGEDTEEEEDEDVD<br>SEDKEDDSEAALGEAESDPHPSHPDQRAHFRGWGYRPGKETEEEEAAEDWGEAEPCPFRVAIYVPGEKPPPPWAPPRLPLRLQRRLKRPETPTHDPDPETPLKARKVRFSEKVTVHFLAVWAGPAQAARQ<br>GPWEQLARDRSRFARRIAQAQEELSPCLTPAARARAWARLRNPPLAPIPALTQTLPSVDGSEFPAGNHHHHHH*	UK2260

Table S2. Plasmids used in this study

Lab number	Lab name	Description	Abbreviation	Reference
**UK105**	eIF2a-NM_pET30a	His6-tagged human eIF2a 1-185 pET-30a(+)"	eIF2a	PMID 15341733
**UK168**	PerkKD-pGEX4T-1	Bacterial expression plasmid for mouse PERK kinase domain	PERK	PMID 9930704
**UK622**	PGV_PP1G_1-323_V1	Bacterial expression plasmid for full-length PP1 phosphatase catalytic domain	PP1G	PMID 25774600
**UK1920***	huPPP1R15A_533_624_malE_pGEX_TEV_AviTag (MP1)	Bacterial expression plasmid for N-tern AviTagged human GADD34 533-624	R15A533-624-MBP	PMID: 28447936
**UK1921***	huPPP1R15A_325_636_malE_pGEX_TEV_AviTag (MP4	Bacterial expression plasmid for N-tern AviTagged human GADD34 325-624	R15A325-636-MBP	PMID: 28447936
**UK2258***	huPPP1R15A_325-636_pMAL-c5X-H6 (AB)**	Bacterial expression plasmid for MBP_1R15A(325-636)-H6	MBP~R15A325-636	PMID: 28759048
**UK2260***	MBP_huPPP1R15A_325-636_H6_pMAL-c5X-His	Bacterial expression plasmid for MBP_R15A(325-636)-H6	MBP-R15A325-636	This study
**UK2261***	MBP_huPPP1R15A_325-512_H6_pMAL-c5X-His	Bacterial expression plasmid for MBP_R15A(325-512)-H6 (N-term)	MBP-R15A325-512	This study
**UK2264**	PP1A_7-330_RP1B (MPC)	Bacterial expression plasmid for H6-TEV-rabbit PP1A 7-330 Peti lab (Addgene Plasmid# 26566)	PP1a	PMID: 18992256

*The sequence of the proteins encoded by these plasmids is available as Table S1

**A gift from Anne Bertolotti's laboratory

Table S3. Number of repeats for the different experiments

Figures		Repeats
1	a	n/a
	b	2
	c	2
2	a	3
	b	3
	c	3
3	a	3
	b	3
4	a	2
	b	2
5	a	3
	b	2
6	a	3
	b	2
7	a	n/a
S1	a	1
	b	1
S2	a	3
	b	1
S3	a	n/a
	b	1
S4	a	2
	b	1
	c	2
